# Associações independentes e agrupadas da atividade física e do comportamento sedentário com a incontinência urinária: dados de mulheres adultas e idosas do ELSI-Brasil

**DOI:** 10.1590/0102-311XPT159125

**Published:** 2026-03-02

**Authors:** Damiana Lima Costa, Enaiane Cristina Menezes, Felipe Fank, Darlan Laurício Matte, Giovana Zarpellon Mazo

**Affiliations:** 1 Universidade do Estado de Santa Catarina, Florianópolis, Brasil.; 2 Universidade Federal de Alagoas, Maceió, Brasil.

**Keywords:** Inatividade Física, Estilo de Vida, Envelhecimento, Saúde da Mulher, Fatores de Risco, Physical Inactivity, Life Style, Aging, Women’s Health, Risk Factors, Inactividad Física, Estilo de Vida, Envejecimento, Salud de la Mujer, Factores de Riesgo

## Abstract

A incontinência urinária é uma condição prevalente entre mulheres idosas, impactando negativamente a qualidade de vida e gerando custos elevados ao sistema de saúde. A atividade física e o comportamento sedentário podem estar associados à incontinência urinária, mas essa relação ainda é pouco explorada. O objetivo deste estudo foi verificar a associação entre diferentes intensidades de atividade física e comportamento sedentário com a ocorrência de incontinência urinária em mulheres adultas e idosas brasileiras. A amostra foi composta por 4.020 mulheres com 50 anos ou mais, participantes da primeira onda (2015-2016) do *Estudo Longitudinal da Saúde dos Idosos Brasileiros* (ELSI-Brasil). A atividade física moderada apresentou associação com menor prevalência de incontinência urinária, com razão de prevalência (RP) variando de 0,66 a 0,73, enquanto a atividade física vigorosa mostrou associação com maior prevalência de incontinência urinária (RP de 1,69 a 1,92). O comportamento sedentário igual ou superior a 6 horas diárias também esteve associado à maior prevalência de incontinência urinária (RP de 1,54 a 1,66). Mulheres com níveis suficientes de atividade física e baixo comportamento sedentário apresentaram redução de aproximadamente 33% a 35% na prevalência de incontinência urinária (RP de 0,65 a 0,67), independentemente de fatores sociodemográficos e físicos. Conclui-se que manter níveis suficientes de atividade física e reduzir o tempo em comportamentos sedentários estão associados à menor prevalência de incontinência urinária em mulheres adultas e idosas.

## Abstract (en)

Urinary incontinence is a prevalent condition in older women, negatively impacting their quality of life and generating high costs for health systems. Physical activity and sedentary behavior may be associated with urinary incontinence but this relationship remains scarcely explored. This study aimed to assess the association between physical activity intensity and sedentary behavior and urinary incontinence in Brazilian adult and older women. The sample consisted of 4,020 women aged 50 years or older who participated in the first wave (2015-2016) of the *Brazilian Longitudinal Study of Aging* (ELSI-Brazil). Moderate physical activity was associated with a lower prevalence of urinary incontinence, with a prevalence ratio (PR) from 0.66 to 0.73, whereas vigorous physical activity was associated with a higher prevalence of urinary incontinence (PR from 1.69 to 1.92). Sedentary behavior equal to or exceeding six hours per day was also associated with a higher prevalence of urinary incontinence (PR from 1.54 to 1.66). Women with sufficient levels of physical activity and low sedentary behavior showed a reduction of about 33-35% in their prevalence of urinary incontinence (PR from 0.65 to 0.67), regardless of sociodemographic and physical factors. Maintaining sufficient levels of physical activity and reducing sedentary behaviors are associated with a lower prevalence of urinary incontinence in adult and older women.

## Abstract (es)

La incontinencia urinaria es una afección prevalente entre mujeres mayores, que repercute negativamente en su calidad de vida y supone elevper capitaados costos para el sistema de salud. La actividad física y el comportamiento sedentario pueden estar asociados a la incontinencia urinaria, pero esta relación aún ha sido poco explorada. El objetivo de este estudio fue verificar la asociación entre diferentes intensidades de actividad física y comportamiento sedentario con la incidencia de incontinencia urinaria en mujeres brasileñas adultas y mayores. La muestra estuvo compuesta por 4.020 mujeres de 50 años o más que participaron en la primera ola (2015-2016) del *Estudio Longitudinal de Salud de los Ancianos Brasileños* (ELSI-Brasil). La actividad física moderada se asoció a una menor prevalencia de incontinencia urinaria, con una razón de prevalencia (RP) que osciló entre 0,66 y 0,73, mientras que la actividad física vigorosa se asoció a una mayor prevalencia de incontinencia urinaria (RP entre 1,69 a 1,92). El comportamiento sedentario igual o mayor que 6 horas diarias también se asoció a una mayor prevalencia de incontinencia urinaria (RP entre 1,54 a 1,66). Las mujeres con niveles suficientes de actividad física y bajo comportamiento sedentario mostraron una reducción de aproximadamente un 33% a un 35% en la prevalencia de incontinencia urinaria (RP entre 0,65 a 0,67), independientemente de los factores sociodemográficos y físicos. Se concluye que mantener niveles suficientes de actividad física y reducir el tiempo dedicado a comportamientos sedentarios está asociado a una menor prevalencia de incontinencia urinaria en mujeres adultas y mayores.

## Introdução

A incontinência urinária caracterizada pela perda involuntária de urina ^1^, é uma condição prevalente que afeta principalmente as mulheres em fases mais avançadas da vida ^2^. Os tipos mais comuns de incontinência urinária incluem a incontinência urinária de esforço, de urgência e mista ^1^. Globalmente, mais de 200 milhões de pessoas sofrem de incontinência urinária, com uma prevalência entre 15% e 52%, predominantemente em mulheres ^3^. No contexto brasileiro, a prevalência de incontinência urinária varia de 26,2 a 60%, sendo mais prevalente em mulheres idosas ^4^ ^,^ ^5^ ^,^ ^6^ ^,^ ^7^.

A incontinência urinária não apenas compromete a qualidade de vida dos indivíduos afetados, impactando suas rotinas diárias e diminuindo sua autoestima devido ao constrangimento associado ^8^, mas também impõe um ônus significativo aos sistemas de saúde ^9^. Estima-se que anualmente ela resulte em custos econômicos diretos e indiretos de aproximadamente mais de USD 7 bilhões em países ocidentais, incluindo despesas com tratamento médico e admissão em instituições de longa permanência ^10^.

Em relação ao assoalho pélvico, estudos indicam que exercícios de baixo a médio impacto podem prevenir disfunções do assoalho pélvico, especialmente a incontinência urinária, ao fortalecer os músculos do assoalho pélvico e promover contrações reflexas durante o aumento da pressão intra-abdominal ^11^ ^,^ ^12^. No entanto, os exercícios de alto impacto são considerados um fator de risco para incontinência urinária, pois podem comprometer os mecanismos de continência ao aumentar a pressão intra-abdominal e sobrecarregar o assoalho pélvico, resultando em seu enfraquecimento ^13^.

A prática regular de atividade física pode ser uma estratégia eficaz para prevenir e minimizar os efeitos da incontinência urinária durante o envelhecimento, uma vez que a atividade física contribui para a manutenção do peso corporal e a prevenção da obesidade, reduzindo a pressão intra-abdominal crônica que pode enfraquecer aper capitas estruturas de suporte pélvico ^14^ ^,^ ^15^. No entanto, o constrangimento causado por episódios acidentais de perda urinária pode levar ao afastamento de situações sociais, criando uma barreira para a prática regular de exercícios ^16^ e resultando na redução dos níveis de atividade física entre idosas com queixas urinárias, o que, por sua vez, pode limitar sua funcionalidade ^17^. 

Além dos baixos níveis de atividade física, o comportamento sedentário emerge como um fator relevante e distinto na determinação da saúde. O comportamento sedentário é definido como qualquer atividade realizada durante o período de vigília com gasto energético ≤ 1,5 METs (equivalente metabólico), em posição sentada, reclinada ou deitada ^18^. Evidências apontam que o comportamento sedentário prolongado está associado a maior mortalidade por todas as causas, doenças cardiovasculares, diabetes tipo 2 e declínio funcional ^19^. Estudos recentes destacam que altos níveis de atividade física não compensam integralmente os efeitos deletérios do comportamento sedentário prolongado, indicando que a combinação de atividade física adequada e baixo comportamento sedentário é mais benéfica à saúde do que níveis elevados de atividade física isoladamente ^20^ ^,^ ^21^.

Embora a atividade física habitual seja eficaz na prevenção e no manejo da incontinência urinária, a literatura ainda não é consistente sobre a relação entre o comportamento sedentário, atividade física e a incontinência urinária em mulheres idosas. Ainda há um grande desconhecimento sobre como um estilo de vida sedentário, que tem um impacto clinicamente significativo na saúde, afeta o risco e a gravidade dessa condição ^22^.

Devido à alta prevalência de incontinência urinária e suas consequências no padrão de atividade física, este problema exerce um impacto significativo na saúde das mulheres, afetando os níveis físico, mental e social. No entanto, existem lacunas na literatura atual sobre a relação entre comportamento sedentário, níveis de atividade física e incontinência urinária, especialmente em idosas brasileiras. A necessidade de estudos específicos nesta população ressalta a carência de pesquisas que explorem essas interações de forma detalhada. Assim, este estudo tem como objetivo verificar a associação entre diferentes intensidades de atividade física e comportamento sedentário com o risco de incontinência urinária em mulheres adultas e idosas brasileiras.

## Métodos

### Design do estudo e amostra

Trata-se de um estudo transversal baseado em dados da primeira onda (2015-2016) do *Estudo Longitudinal da Saúde dos Idosos Brasileiros* (ELSI-Brasil), representativo da população brasileira com 50 anos ou mais. O ELSI-Brasil utiliza amostragem probabilística por múltiplos estágios e estratificação por conglomerados em áreas urbanas e rurais, abrangendo 70 municípios das cinco regiões do país. Informações detalhadas sobre o desenho e a representatividade da amostra estão disponíveis em https://elsi.cpqrr.fiocruz.br/.

**Figura 1:**
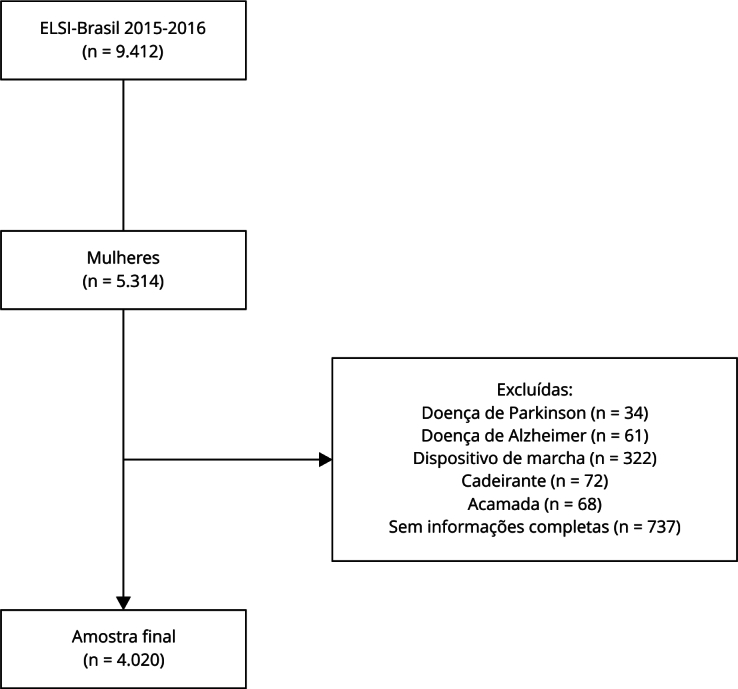
Processo de seleção da amostra. *Estudo Longitudinal da Saúde dos Idosos Brasileiros* (ELSI-Brasil), 2015-2016.

Para o presente estudo, foram incluídas 4.020 mulheres com 50 anos ou mais, com dados completos das variáveis de interesse. Foram excluídas participantes com doença de Parkinson (n = 34), doença de Alzheimer (n = 61), uso de dispositivos auxiliares de marcha (n = 322), cadeirantes (n = 72) e acamadas (n = 68). O fluxograma do processo de seleção da amostra é apresentado na Figura 1, detalhando as etapas de inclusão e exclusão das participantes. O estudo foi aprovado pelo Comitê de Ética em Pesquisa da instituição coordenadora do ELSI-Brasil (Fundação Oswaldo Cruz, CAAE: 34.649.814.3.0000.5091) e todos os participantes assinaram o termo de consentimento livre e esclarecido.

### Variável desfecho

A incontinência urinária foi identificada pela pergunta: “No último mês, alguma vez o(a) Sr(a) perdeu o controle da urina sem querer?”. As respostas foram categorizadas em “sim” (presença de incontinência urinária) e “não” (ausência de incontinência urinária ).

### Variáveis de exposição

As variáveis de exposição foram a atividade física e o comportamento sedentário, obtidas por meio de autorrelato conforme o protocolo do ELSI-Brasil. Para este estudo, a soma semanal das atividades foi calculada para cada uma das intensidades (leve, moderada e vigorosa) e classificada de acordo com as recomendações do *Guia de Atividade Física para a População Brasileira* ^23^: (a) atividade física leve (0 minutos, 1-149 minutos, 150-299 minutos, ≥ 300 minutos); (b) atividade física moderada (0 minutos, 1-149 minutos, 150-299 minutos, ≥ 300 minutos); e (c) atividade física vigorosa (0 minutos, 1-74 minutos, 75-149, ≥ 150 minutos). Essa classificação foi utilizada para analisar as associações isoladas das diferentes intensidades da atividade física com o desfecho.

Além disso, também foi avaliada a atividade física moderada e vigorosa considerando as recomendações do *Guia de Atividade Física para a População Brasileira* ^23^, de pelo menos 150 minutos de atividade física moderada, ou 75 minutos de intensidade vigorosa por semana, ou qualquer combinação equivalente das duas intensidades. A soma destas atividades foi categorizada em “atinge as recomendações” e “não atinge as recomendações” e foi empregada para analisar as associações dos comportamentos agrupados com o desfecho. O tempo total em comportamento sedentário foi determinado com base na média ponderada do tempo sentado em um dia de semana e em um dia de final de semana ((tempo na semana × 5) + (tempo no final de semana × 2))/7). Na sequência, o tempo diário despendido em comportamento sedentário foi categorizado em: (a) < 3 horas; (b) entre 3 e 6 horas; e (c) > 6 horas ^24^, sendo esta última categorização considerada o ponto de corte de alto ou baixo comportamento sedentário nas análises agrupadas.

De acordo com as classificações da atividade física e do comportamento sedentário, as participantes foram agrupadas em quatro categorias: (1) atividade física suficiente/baixo comportamento sedentário − atingem as recomendações de atividade física e passam menos de seis horas diárias em comportamento sedentário; (2) atividade física suficiente/alto comportamento sedentário − atingem as recomendações de atividade física e passam mais de seis horas diárias em comportamento sedentário; (3) atividade física insuficiente/baixo comportamento sedentário − não atingem as recomendações de atividade física e passam menos de seis horas diárias em comportamento sedentário; e (4) atividade física insuficiente/alto comportamento sedentário − não atingem as recomendações de atividade física e passam mais de seis horas diárias em comportamento sedentário.

### Variáveis de ajuste

As variáveis de confundimento incluíram os dados sociodemográficos, hábitos, condições de saúde e características físicas. As informações sociodemográficas englobaram: idade (50-59, 60-69, 70-79 e ≥ 80 anos); estado conjugal (com ou sem companheiro(a)); escolaridade, registrada com o número de anos de estudo (nenhum, 1-4, 5-8 e ≥ 9); cor da pele autorreferida (branca, preta, parda, amarela e indígena), agrupada em branca ou não branca; renda domiciliar *per capita* (em reais); e filhos (sim ou não). As variáveis sobre hábitos e condições de saúde incluíram: menstruação (sim ou não); tratamento hormonal (faz atualmente, já fez, mas não faz mais e não); histerectomia (sim ou não); etilismo (nunca, menos de uma vez por mês e uma vez ou mais por mês); tabagismo (diariamente, menos que diariamente e nunca fumou); percepção de saúde agrupada em positiva (excelente, muito boa, boa) ou negativa (regular, ruim e muito ruim); hipertensão arterial (sim ou não); diabetes (sim ou não); depressão (sim ou não); e cognição. As características físicas abrangeram a circunferência de cintura, a força de preensão manual e a velocidade da marcha.

### Análise estatística

A análise descritiva das características sociodemográficas e de saúde das participantes foi realizada por meio da frequência relativa e de seus respectivos intervalos de 95% de confiança (IC95%), ambos calculados levando em consideração o delineamento da amostra e os pesos amostrais. Os dados foram analisados tanto para a amostra total quanto segundo as intensidades da atividade física. As possíveis associações simples entre a incontinência urinária e as variáveis de confusão foram avaliadas por meio do teste de qui-quadrado de Pearson, com correção de Rao-Scott para amostras complexas.

Para investigar as associações entre as variáveis de exposição (as diferentes intensidades da atividade física, o comportamento sedentário e o agrupamento) e a variável desfecho, agrupada em “com” e “sem” incontinência urinária, foram determinadas as razões de prevalência (RP) e seus respectivos IC95%. Foram testados quatro modelos. O modelo A foi ajustado por todas as variáveis que se associaram ao desfecho, o modelo B ajustado de acordo com as características sociodemográficas, o modelo C ajustado de acordo com hábitos e condições de saúde e o modelo D ajustado de acordo com as características físicas. Essa estratégia de ajuste por blocos temáticos permite avaliar separadamente o impacto de diferentes grupos de variáveis sobre as associações observadas. As análises foram realizadas no software R versão 4.3.3 (http://www.r-project.org), utilizando o pacote *survey*, que considera a estrutura complexa da amostra (pesos atribuídos aos indivíduos e parâmetros amostrais). Todos os resultados levaram em consideração um nível de 5% de significância (p < 0,05).

## Resultados

Participaram deste estudo 4.020 mulheres com 50 anos ou mais de idade que participaram da primeira fase do ELSI-Brasil (2015-2016). A maioria tinha entre 50-59 anos (49,2%), havia estudado entre 1 e 4 anos (38,2%), possuía cor de pele branca (56,6%), companheiro (57,1%) e renda domiciliar *per capita* média de BRL 1.153,50. Além disso, a maioria das participantes relatou não menstruar (92,5%), não fazer uso de tratamento hormonal (73,4%), não ter feito histerectomia (81,1%), não fazer uso de bebidas alcoólicas (79,4%) e nunca ter fumado (86%). Ainda, a maioria referiu não ter tido queda nos últimos 12 meses (75,4%) e tinha uma percepção negativa sobre sua saúde (54,8%). A prevalência geral de incontinência urinária entre as mulheres foi de 7,9% (IC95%: 6,6-9,3) (Tabela 1).

**Tabela 1:** Características sociodemográficas e história clínica das mulheres adultas e idosas brasileiras em relação ao desfecho de incontinência urinária. *Estudo Longitudinal da Saúde dos Idosos Brasileiros* (ELSI-Brasil) 2015-2016, (n = 4.020).

Variáveis	Total (n = 4.020)		Com incontinência urinária (n = 313)		Sem incontinência urinária (n = 3.707)	
	%	IC95%	%	IC95%	%	IC95%
Idade (anos)						
50-59	49,2	45,6-52,7	45,1	37,4-52,7	49,5	45,8-53,2
60-69	30,5	28,4-32,6	24,9	18,9-30,8	31,0	28,7-33,3
70-79	15,2	13,4-16,9	18,5	13,6-23,5	14,9	13,1-16,7
≥ 80	5,1	3,8-5,9	11,5	6,8-14,7	4,6	3,3-5,3
Escolaridade (anos de estudo)						
0	11,7	9,3-14,1	10,0	6,5-13,5	11,8	9,3-14,3
1-4	38,2	35,2-41,2	37,2	31,4-43,0	38,3	35,1-41,4
5-8	21,9	19,2-24,7	22,5	14,9-30,1	21,9	19,2-24,6
≥ 9	28,2	25,3-31,1	30,3	23,7-36,9	28,0	25,1-31,0
Cor da pele						
Branca	56,6	50,4-62,8	53,7	45,7-61,8	56,8	50,4-63,1
Não branca	43,4	37,2-49,6	46,3	38,2-54,3	43,2	36,9-49,5
Estado conjugal						
Com companheiro	57,1	53,9-60,2	56,1	49,4-62,8	57,1	54,0-60,3
Sem companheiro	42,9	39,8-46,1	43,9	37,2-50,6	42,9	39,7-46,0
Filhos (número) [média/erro padrão]	3,3	0,09	3,4	0,20	3,3	0,10
Renda (em reais) [média/erro padrão]	1.153,5	54,2	1.313,3	100,2	1.139,8	54,2
Menstruação						
Sim	7,5	6,6-8,5	5,8	2,7-8,8	7,7	6,7-8,7
Não	92,5	91,5-93,4	94,2	91,2-97,3	92,3	91,3-93,3
Tratamento hormonal						
Faz atualmente	4,8	3,6-6,0	5,0	2,3-7,4	4,8	3,6-6,1
Já fez, mas não faz mais	21,8	19,7-23,8	24,0	18,5-29,6	21,6	19,4-23,8
Não	73,4	71,2-75,6	71,0	65,0-76,9	73,6	71,3-75,9
Histerectomia						
Sim	18,9	17,2-20,7	22,5	17,5-27,5	18,6	16,8-20,4
Não	81,1	79,3-82,8	77,5	72,5-82,5	81,4	79,6-83,2
Etilismo						
Nunca	79,4	77,6-81,3	76,9	70,8-83,1	79,7	77,7-81,6
Menos de uma vez por mês	6,6	5,3-7,8	8,3	5,2-11,4	6,4	5,2-7,7
Uma vez ou mais por mês	14,0	12,2-15,8	14,8	9,0-20,5	13,9	12,0-15,8
Tabagismo						
Diariamente	12,8	11,0-14,5	12,8	8,5-17,1	12,8	10,9-14,7
Menos que diariamente	1,2	0,7-1,6	0,5	0,1-1,1	1,2	0,8-1,7
Nunca fumou	86,0	84,2-87,7	86,7	82,3-91,1	86,0	84,0-87,9
Percepção de saúde						
Positiva	45,2	42,4-48,1	30,8	23,6-38,1	46,5	43,6-49,4
Negativa	54,8	51,9-57,6	69,2	61,9-76,4	53,5	50,6-56,4
Quedas						
Sim	24,6	23,1-26,1	35,4	29,4-41,5	23,7	22,1-25,2
Não	75,4	73,9-76,9	64,6	58,5-70,6	76,3	74,8-77,9
Hipertensão arterial sistêmica						
Sim	54,1	51,8-56,4	60,0	52,7-67,2	53,6	51,2-56,0
Não	45,9	43,6-48,2	40,0	32,8-47,3	46,4	44,0-48,8
Diabetes						
Sim	15,4	13,6-17,2	21,1	16,0-26,3	14,9	13,2-16,7
Não	84,6	82,8-86,4	78,9	73,7-84,0	85,1	83,3-86,8
Doença pulmonar obstrutiva crônica						
Sim	6,3	5,4-7,3	13,1	8,4-17,7	5,8	4,8-6,7
Não	93,7	92,7-94,6	86,9	82,3-91,6	94,2	93,3-95,8
Depressão						
Sim	25,3	22,6-28,0	42,4	36,1-48,7	23,8	21,1-26,5
Não	74,7	72,0-77,4	57,6	51,3-63,9	76,2	73,5-78,9
Circunferência da cintura [média/erro padrão]	91,8	0,42	95,6	1,05	91,6	0,41
Força de preensão manual [média/erro padrão]	21,7	0,21	20,3	0,46	21,8	0,21
Velocidade da marcha						
Fraco	68,4	64,0-72,8	81,1	75,0-87,3	67,6	63,0-72,1
Bom	31,6	27,2-36,0	18,9	12,7-25,0	32,4	27,9-37,0

IC95%: intervalo de 95% de confiança.

A Tabela 2 apresenta os resultados das associações entre diferentes intensidades e níveis de atividade física, comportamento sedentário e incontinência urinária em mulheres adultas e idosas, analisadas por quatro modelos ajustados. Em todos os modelos, a prática de atividade física moderada de até 149 minutos por semana esteve consistentemente associada a uma menor prevalência de incontinência urinária. A magnitude dessa associação variou ligeiramente entre os modelos: modelo A (RP = 0,73; IC95%: 0,53-0,99), modelo B (RP = 0,71; IC95%: 0,51-0,97), modelo C (RP = 0,66; IC95%: 0,48-0,90) e modelo D (RP = 0,70; IC95%: 0,51-0,96). Em resumo, esses resultados indicam que a prática de atividade física moderada está associada a menor prevalência de incontinência urinária, mesmo após o controle por diferentes fatores demográficos, socioeconômicos, hábitos e condições de saúde, além de variáveis físicas (Tabela 2).

**Tabela 2:** Associação entre incontinência urinária, intensidades e níveis da atividade física e o comportamento sedentário em mulheres adultas e idosas brasileiras. *Estudo Longitudinal da Saúde dos Idosos Brasileiros* (ELSI-Brasil), 2015-2016.

Exposição	Total n (%)	Incontinência urinária		Modelo A ajustado	Modelo B ajustado	Modelo C ajustado	Modelo D ajustado
		Sim n (%)	Não n (%)	RP (IC95%)	RP (IC95%)	RP (IC95%)	RP (IC95%)
Atividade física leve (minutos)							
0	745 (19,5)	74 (24,5)	671 (19,0)	1,00	1,00	1,00	1,00
1-149	1.269 (33,2)	95 (31,5)	1.174 (33,3)	0,91 (0,67-1,24)	0,81 (0,60-1,10)	0,81 (0,58-1,12)	0,82 (0,61-1,12)
150-299	572 (15,0)	40 (13,2)	532 (15,1)	0,88 (0,53-1,45)	0,71 (0,44-1,15)	0,80 (0,47-1,29)	0,77 (0,47-1,27)
≥ 300	1.239 (32,3)	93 (30,8)	1.146 (32,6)	0,96 (0,68-1,36)	0,77 (0,55-1,07)	0,82 (0,59-1,15)	0,87 (0,62-1,21)
Atividade física moderada (minutos)							
0	1.424 (36,2)	1.299 (35,8)	125 (40,6)	1,00	1,00	1,00	1,00
1-149	924 (23,5)	864 (23,8)	60 (19,5)	0,73 (0,54-0,99)	0,71 (0,51-0,97)	0,66 (0,48-0,90)	0,70 (0,51-0,96)
150-299	436 (11,1)	402 (11,1)	34 (11,0)	0,90 (0,60-1,34)	0,83 (0,54-1,28)	0,77 (0,52-1,14)	0,83 (0,55-1,26)
≥ 300	1.151 (29,2)	1.062 (29,3)	89 (28,9)	0,94 (0,70-1,30)	0,87 (0,65-1,17)	0,81 (0,62-1,09)	0,89 (0,66-1,19)
Atividade física vigorosa (minutos)							
0	2.909 (72,4)	2.692 (72,6)	217 (69,3)	1,00	1,00	1,00	1,00
1-74	333 (8,3)	306 (8,3)	27 (8,6)	1,24 (0,79-1,92)	1,29 (0,84-1,97)	1,14 (0,71-1,79)	1,19 (0,78-1,81)
75-149	214 (5,3)	192 (5,2)	22 (7,0)	1,92 (1,21-3,03)	1,81 (1,14-2,87)	1,69 (1,06-2,72)	1,75 (1,09-2,81)
≥ 150	564 (14,0)	517 (13,9)	47 (15,1)	1,23 (0,89-1,71)	1,19 (0,87-1,64)	1,11 (0,83-1,50)	1,19 (0,86-1,63)
Atividade física moderada a vigorosa (minutos)							
0	1.303 (33,4)	1.194 (33,2)	109 (36,0)	1,00	1,00	1,00	1,00
1-149	827 (21,2)	773 (21,5)	54 (17,8)	0,86 (0,59-1,24)	0,85 (0,58-1,26)	0,76 (0,53-1,11)	0,83 (0,56-1,23)
≥ 150	1.774 (45,4)	1.634 (45,3)	140 (46,2)	1,00 (0,75-1,34)	0,92 (0,69-1,22)	0,82 (0,63-1,07)	0,92 (0,70-1,22)
Comportamento sedentário (horas)							
< 3	2.196 (56,3)	2.053 (57,0)	143 (47,2)	1,00	1,00	1,00	1,00
3 e 6	1.418 (36,3)	1.292 (35,9)	126 (41,6)	1,16 (0,85-1,54)	1,21 (0,91-1,61)	1,18 (0,85-1,62)	1,26 (0,95-1,67)
> 6	289 (7,4)	255 (7,1)	34 (11,2)	1,38 (0,95-1,98)	1,66 (1,12-2,44)	1,54 (1,06-2,21)	1,56 (1,10-2,21)

IC95%: intervalo de 95% de confiança; RP: razão de prevalência.

Modelo A (p < 0,05): idade, filhos, comportamento sedentário/atividade física moderada a vigorosa, percepção de saúde, hipertensão arterial sistêmica, diabetes, doença pulmonar obstrutiva crônica, depressão, circunferência da cintura, força de preensão manual, velocidade da marcha; modelo B (sociodemográficas): idade, estado civil, escolaridade, raça, renda e filhos; modelo C (hábitos e condições de saúde): menstruação, tratamento hormonal, histerectomia, comportamento sedentário/atividade física moderada a vigorosa, bebida alcoólica, tabagismo, percepção de saúde, hipertensão arterial sistêmica, diabetes, doença pulmonar obstrutiva crônica, depressão, cognição; modelo D (físicas): circunferência da cintura, força de preensão manual e velocidade da marcha.

Em contraste, ao analisar a atividade física vigorosa, identificou-se uma associação com a incontinência urinária em todos os modelos ajustados (Tabela 2): modelo A: RP = 1,92 (IC95%: 1,21-3,03); modelo B: RP = 1,81 (IC95%: 1,14-2,87); modelo C: RP = 1,69 (IC95%: 1,06-2,72); e modelo D: RP = 1,75 (IC95%: 1,09-2,81). Esses resultados sugerem que a prática de atividades físicas vigorosas, entre 75-149 minutos por semana, está associada a maior prevalência de incontinência urinária, independentemente dos fatores sociodemográficos, hábitos de saúde e variáveis físicas.

Em relação ao comportamento sedentário, os resultados indicam que passar mais de 6 horas por dia nesse comportamento está associado a uma maior prevalência de incontinência urinária, especialmente quando ajustado para variáveis sociodemográficas, hábitos de saúde e variáveis físicas (Tabela 2). Embora o Modelo A não tenha mostrado associação significativa (RP = 1,38; IC95%: 0,96-1,98), os modelos B (RP = 1,66; IC95%: 1,12-2,44), C (RP = 1,54; IC95%: 1,06-2,21) e D (RP = 1,56; IC95%: 1,10-2,21) evidenciaram uma associação consistente e significativa entre maior tempo em comportamento sedentário e a prevalência de incontinência urinária.

A Tabela 3 apresenta os resultados das associações entre o agrupamento dos níveis de atividade física e o comportamento sedentário com a incontinência urinária, analisados por quatro modelos ajustados. Os resultados indicam que tanto a atividade física insuficiente quanto a suficiente, quando combinadas com baixo comportamento sedentário, estão associadas a uma menor prevalência de incontinência urinária. Especificamente, mulheres com atividade física insuficiente e baixo comportamento sedentário apresentaram uma RP 31% menor (IC95%: 0,48-0,98) no modelo ajustado para variáveis sociodemográficas e 30% menor (IC95%: 0,49-0,99) no modelo ajustado por variáveis físicas. Da mesma forma, aquelas com atividade física suficiente e baixo comportamento sedentário apresentaram uma RP 33% menor (IC95%: 0,46-0,96) no modelo sociodemográfico e 35% menor (IC95%: 0,45-0,94) no modelo ajustado para condições físicas. Esses achados sugerem que manter-se fisicamente ativa e reduzir o tempo em comportamento sedentário estão associados a uma menor prevalência de incontinência urinária, independentemente dos fatores sociodemográficos e físicos considerados.

**Tabela 3:** Associação entre o agrupamento da atividade física e do comportamento sedentário com a incontinência urinária em mulheres adultas e idosas brasileiras. *Estudo Longitudinal da Saúde dos Idosos Brasileiros* (ELSI-Brasil), 2015-2016.

Comportamentos	Total n (%)	Incontinência urinária		Modelo A ajustado	Modelo B ajustado	Modelo C ajustado	Modelo D ajustado
		Sim n (%)	Não n (%)	RP (IC95%)	RP (IC95%)	RP (IC95%)	RP (IC95%)
Atividade física insuficiente/Alto comportamento sedentário	259 (6,6)	229 (6,4)	30 (9,9)	1,00	1,00	1,00	1,00
Atividade física insuficiente/Baixo comportamento sedentário	1.870 (47,9)	1.737 (48,2)	133 (43,9)	0,76 (0,54-1,11)	0,69 (0,48-0,98)	0,71 (0,48-1,06)	0,70 (0,49-0,99)
Atividade física suficiente/Alto comportamento sedentário	203 (5,2)	184 (5,1)	19 (6,3)	0,64 (0,31-1,33)	0,63 (0,32-1,22)	0,58 (0,28-1,22)	0,63 (0,32-1,22)
Atividade física suficiente/Baixo comportamento sedentário	1.571 (40,3)	1.450 (40,3)	121 (39,9)	0,78 (0,54-1,14)	0,67 (0,46-0,96)	0,67 (0,45-1,00)	0,65 (0,45-0,94)

IC95%: intervalo de 95% de confiança; RP: razão de prevalência.

Modelo A (p < 0,05): idade, filhos, percepção de saúde, hipertensão arterial sistêmica, diabetes, doença pulmonar obstrutiva crônica, depressão, circunferência da cintura, força de preensão manual, velocidade da marcha; modelo B (sociodemográficas): idade, sexo, renda, estado civil, raça, escolaridade; modelo C (hábitos e condições de saúde): bebida alcoólica, tabagismo, percepção de saúde, hipertensão arterial sistêmica, diabetes, doença pulmonar obstrutiva crônica, depressão, cognição; modelo D (físicas): circunferência da cintura, força de preensão manual e velocidade da marcha.

## Discussão

A presente investigação observou associações entre diferentes intensidades de atividade física e comportamento sedentário com a ocorrência de incontinência urinária em mulheres adultas e idosas brasileiras. Verificou-se que a prática de atividade física moderada está associada a menor probabilidade de incontinência urinária, enquanto a atividade física vigorosa está relacionada a maior prevalência dessa condição. Além disso, permanecer mais de seis horas em comportamento sedentário esteve associado a maior ocorrência de incontinência urinária. Por fim, a combinação de níveis adequados de atividade física e baixo comportamento sedentário mostrou-se associada a menor probabilidade de incontinência urinária.

As diretrizes da Organização Mundial da Saúde (OMS) recomendam que idosos pratiquem de 150 a 300 minutos de atividade física moderada ou 75 a 150 minutos de atividade física vigorosa semanalmente ^25^. A atividade física é essencial para essa faixa etária, pois se associa a melhorias nas habilidades motoras, força muscular, mobilidade, flexibilidade e cognição, contribuindo para uma melhor qualidade de vida ^26^. Além disso, manter-se fisicamente ativo durante o envelhecimento relaciona-se a benefícios gerais para a saúde, podendo estar vinculado à prevenção de condições como a incontinência urinária, especialmente entre mulheres idosas ^27^.

Embora a prática regular de atividade física seja considerada benéfica na prevenção primária e secundária da incontinência urinária, sua relação com as disfunções do assoalho pélvico é complexa e influenciada por múltiplos fatores de mediação e confusão ^28^. Fatores como peso corporal, força muscular do assoalho pélvico, nível de condicionamento físico e pressão intra-abdominal podem modificar a associação entre diferentes intensidades de exercício e a ocorrência de sintomas urinários ^29^.

Além disso, mulheres com incontinência urinária frequentemente reduzem ou interrompem a prática de atividades devido ao desconforto e constrangimento associados aos sintomas ^30^. A gravidade desses sintomas está relacionada ao aumento do sedentarismo, evidenciando o impacto negativo da incontinência na adesão à prática de atividades físicas ^30^. Essa dinâmica destaca a complexidade da relação entre engajamento em atividades físicas e a incontinência, mostrando como essa condição pode dificultar a manutenção de um estilo de vida ativo para as mulheres.

Diversos estudos têm evidenciado os efeitos benéficos da atividade física moderada sobre a função do assoalho pélvico. Faleiro et al. ^31^ mostraram que caminhadas rápidas e exercícios leves estão associados à menor prevalência de incontinência urinária em mulheres mais velhas. De forma semelhante, Kikuchi et al. ^32^ identificaram que altos níveis de atividade física estavam relacionados à menor prevalência autorrelatada de incontinência urinária entre idosos da comunidade. Resultados semelhantes foram observados por Kim et al. ^33^, que verificaram que atividades moderadas de lazer ou trabalho estavam associadas à menor probabilidade de incontinência de esforço, urgência ou mista. Esses achados reforçam o papel protetor da atividade física moderada em diferentes faixas etárias e contextos.

Por outro lado, níveis elevados de atividade física vigorosa têm sido associados a maior ocorrência de sintomas urinários em populações variadas ^34^ ^,^ ^35^ ^,^ ^36^. Um estudo transversal avaliou 503 esportistas (média de 21,1±3,6 anos) que praticam exercícios de alto impacto. A intensidade da atividade física, medida pelo *Questionário Internacional de Atividade Física* (IPAQ, acrônimo em inglês), mostrou valores significativamente maiores de atividade vigorosa no grupo de desportistas com perda urinária ^34^.

Complementando esses achados, Kuutti et al. ^35^ investigaram a relação entre atividade física ao longo da vida e sintomas de distúrbios do assoalho pélvico em 1.098 mulheres finlandesas de 47 a 55 anos. Embora a prática atual de atividade física não tenha sido associada a esses sintomas, mulheres com histórico de esportes competitivos na juventude apresentaram um risco significativamente maior de incontinência urinária de urgência. O estudo reforça a hipótese de que atividades físicas extenuantes durante a juventude podem aumentar o risco de incontinência urinária na meia-idade.

Adicionalmente, Parr et al. ^36^ exploraram a prevalência de incontinência urinária de esforço em 209 atletas universitárias nulíparas, revelando que essas mulheres apresentam taxas significativamente mais altas de incontinência urinária de esforço em comparação com aquelas que não praticam atividades físicas. O estudo destacou que as atletas envolvidas em atividades físicas vigorosas durante mais de cinco dias por semana e que praticam esportes de alto impacto têm pontuações elevadas no *International Consultation on Incontinence Questionnaire − Short Form* (ICIQ-SF, *Questionário Internacional de Consulta sobre Incontinência* - *Versão Resumida*), corroborando a ideia de que a intensidade e o tipo de atividade física podem influenciar o risco de incontinência urinária.

Em relação ao comportamento sedentário, observou-se que mulheres que permanecem mais de seis horas por dia em atividades sedentárias apresentam maior probabilidade de incontinência urinária. Revisões recentes mostram que mulheres idosas com incontinência urinária passam, em média, de 5 a 8 horas por dia em comportamento sedentário, um tempo geralmente maior do que o observado em mulheres da mesma faixa etária sem incontinência urinária ^22^. Complementando essas descobertas, o estudo de Di et al. ^37^ revelou que um tempo sentado prolongado, definido como 7 horas ou mais por dia, está associado a sintomas de incontinência urinária de urgência, bem como a sintomas de incontinência urinária de esforço nas mulheres. Além disso, pesquisas anteriores apontaram que o uso de telas por ≥ 6 horas diárias está relacionado ao desenvolvimento de doenças da bexiga, enquanto uma duração de ≥ 8 horas se associa a distúrbios do sistema geniturinário ^38^.

O principal achado desta pesquisa refere-se à interação entre a atividade física e o comportamento sedentário. Os resultados indicam que, embora a prática adequada de atividade física esteja relacionada a menor probabilidade de incontinência urinária, o tempo prolongado em comportamento sedentário pode atenuar esses benefícios. Assim, mesmo entre mulheres que atingem as recomendações de atividade física, permanecer sentadas por longos períodos está associado a piores desfechos urinários. Esses achados reforçam a necessidade de estratégias combinadas, que aliem o aumento da atividade física moderada à redução do comportamento sedentário ao longo do dia, como pausas ativas e incentivo à mobilidade cotidiana, a fim de maximizar os benefícios para a saúde pélvica.

Este estudo apresenta algumas limitações que devem ser consideradas na interpretação dos resultados. Primeiramente, o delineamento transversal impede estabelecer relações de causa e efeito entre as variáveis analisadas. Além disso, todas as informações foram obtidas por meio de autorrelato, o que pode introduzir viés de memória e desejabilidade social, especialmente em relação à prática de atividade física, comportamento sedentário e sintomas urinários.

Outro ponto importante refere-se à definição do desfecho, obtido por meio de uma única questão que incluía tanto a perda involuntária de urina quanto de fezes. Esse parâmetro pode ter comprometido a especificidade da incontinência urinária e levado à superestimação da prevalência, devendo ser interpretada com cautela. No entanto, essa medida é coerente com instrumentos amplamente empregados em inquéritos populacionais e reflete as limitações inerentes à base de dados do ELSI-Brasil.

Adicionalmente, a ausência de informações sobre os subtipos de incontinência urinária (de esforço, de urgência ou mista) representa outra limitação, uma vez que cada subtipo possui mecanismos fisiológicos distintos. Enquanto a incontinência de esforço tende a estar relacionada ao aumento da pressão intra-abdominal durante atividades vigorosas, a incontinência de urgência pode estar mais ligada a alterações neuromusculares ou comportamentais ^39^. Estudos futuros que considerem essa diferenciação poderão aprofundar a compreensão das associações observadas.

Por fim, não foram utilizadas medidas objetivas para aferição da atividade física e do comportamento sedentário, como acelerometria, nem foram considerados fatores clínicos adicionais, como histórico obstétrico, uso de medicamentos ou avaliação funcional do assoalho pélvico, que poderiam atuar como potenciais confundidores ou mediadores das associações observadas. Estudos longitudinais com medidas mais específicas e detalhadas são necessários para aprofundar a compreensão desses achados e embasar estratégias de prevenção e manejo da incontinência urinária em mulheres.

## Conclusão

Os resultados do estudo demonstraram que a prática de atividade física moderada esteve associada à menor probabilidade de incontinência em mulheres adultas e idosas brasileiras, enquanto níveis de atividade física vigorosa mostraram relação oposta. Além disso, o comportamento sedentário prolongado (≥ 6 horas/dia) foi identificado como um fator de risco relevante.

Embora o delineamento transversal não permita estabelecer relações de causalidade, os achados sugerem que manter níveis adequados de atividade física e reduzir o tempo em comportamento sedentário podem representar estratégias promissoras para a prevenção e o manejo da incontinência urinária. Nesse contexto, destaca-se o papel da fisioterapia pélvica − especialmente o treinamento da musculatura do assoalho pélvico −, aliada à promoção da atividade física moderada e à redução do tempo em comportamento sedentário, como uma abordagem não invasiva, segura e custo-efetiva para a prevenção e o tratamento da incontinência urinária em mulheres adultas e idosas. Tais evidências reforçam a importância de políticas públicas e programas de saúde que incentivem estilos de vida ativos e sustentáveis, visando a promoção de um envelhecimento mais saudável e a melhoria da qualidade de vida.

## Data Availability

Os dados de pesquisa estão disponíveis mediante solicitação à autora de correspondência.

## References

[1] Haylen BT, Ridder D, Freeman RM, Swift SE, Berghmans B, Lee J (2010). An International Urogynecological Association (IUGA)/International Continence Society (ICS) joint report on the terminology for female pelvic floor dysfunction.. Int Urogynecol J.

[2] Faltin D-L (2009). Epidemiology and definition of female urinary incontinence.. J Gynecol Obstet Biol Reprod (Paris).

[3] Norton P, Brubaker L (2006). Urinary incontinence in women.. Lancet.

[4] Jesus Menezes MA, Hashimoto SY, Gouveia Santos VLC (2009). Prevalence of urinary incontinence in a community sample from the city of São Paulo.. J Wound Ostomy Continence Nurs.

[5] Marques LP, Schneider IJC, d’Orsi E, Antes DL, Ramos LR, Kuschnir MCC (2015). Fatores demográficos, condições de saúde e hábitos de vida associados à incontinência urinária em idosos de Florianópolis, Santa Catarina.. Rev Bras Epidemiol.

[6] Reigota RB, Pedro AO, Parreira BDM, Pinto-Neto AM (2016). Prevalence of urinary incontinence and its association with multimorbidity in women aged 50 years or older: a population-based study.. Neurourol Urodyn.

[7] Tamanini JTN, Lebrão ML, Duarte YAO, Santos JLF, Laurenti R (2009). Analysis of the prevalence of and factors associated with urinary incontinence among elderly people in the Municipality of São Paulo, Brazil: SABE Study (Health, Wellbeing and Aging).. Cad Saúde Pública.

[8] Kessler M, Facchini LA, Soares MU, Nunes BP, França SM, Thumé E (2018). Prevalence of urinary incontinence among the elderly and relationship with physical and mental health indicators.. Rev Bras Geriatr Gerontol.

[9] Minassian VA, Drutz HP, Al-Badr A (2003). Urinary incontinence as a worldwide problem.. Int J Gynaecol Obstet.

[10] Milsom I, Coyne KS, Nicholson S, Kvasz M, Chen CI, Wein AJ (2014). Global prevalence and economic burden of urgency urinary incontinence: a systematic review.. Eur Urol.

[11] Bø K (2004). Urinary incontinence, pelvic floor dysfunction, exercise and sport.. Sports Med.

[12] Magaldi CM, Saraiva A, Franciulli PM, Magaldi FM, Moreno M, Miranda MLJ (2018). The influence of physical activity on functional performance and urinary incontinence in elderly women.. Journal of Morphological Sciences.

[13] Virtuoso JF, Mazo GZ, Menezes EC (2011). Urinary incontinence and perineal muscle function in physically active and sedentary elderly women.. Braz J Phys Ther.

[14] Danforth KN, Shah AD, Townsend MK, Lifford KL, Curhan GC, Resnick NM (2007). Physical activity and urinary incontinence among healthy, older women.. Obstet Gynecol.

[15] Townsend MK, Danforth KN, Rosner B, Curhan GC, Resnick NM, Grodstein F (2008). Physical activity and incident urinary incontinence in middle-aged women.. J Urol.

[16] Peterson JA (2008). Minimize urinary incontinence: maximize physical activity in women.. Urol Nurs.

[17] Erekson EA, Ciarleglio MM, Hanissian PD, Strohbehn K, Bynum JP, Fried TR (2015). Functional disability and compromised mobility among older women with urinary incontinence.. Female Pelvic Med Reconstr Surg.

[18] Tremblay MS, Aubert S, Barnes JD, Saunders TJ, Carson V, Latimer-Cheung AE (2017). Sedentary Behavior Research Network (SBRN) - terminology consensus project process and outcome.. Int J Behav Nutr Phys Act.

[19] Rezende LF, Rey-López JP, Matsudo VK, Carmo Luiz O (2014). Sedentary behavior and health outcomes among older adults: a systematic review.. BMC Public Health.

[20] Ekelund U, Tarp J, Steene-Johannessen J, Hansen BH, Jefferis B, Fagerland MW (2019). Dose-response associations between accelerometry measured physical activity and sedentary time and all-cause mortality: systematic review and harmonised meta-analysis.. BMJ.

[21] Stamatakis E, Gale J, Bauman A, Ekelund U, Hamer M, Ding D (2019). Sitting time, physical activity, and risk of mortality in adults.. J Am Coll Cardiol.

[22] Leung WKC, Cheung J, Wong VCC, Tse KKL, Lee RWY, Lam SC (2024). Patterns of sedentary behavior among older women with urinary incontinence and urinary symptoms: a scoping review.. BMC Public Health.

[23] Ministério da Saúde Guia de atividade física para a população brasileira..

[24] Canever JB, Cândido LM, Moreira BS, Danielewicz AL, Cimarosti HI, Lima-Costa MF (2023). A nationwide study on sleep complaints and associated factors in older adults: ELSI-Brazil.. Cad Saúde Pública.

[25] Bull FC, Al-Ansari SS, Biddle S, Borodulin K, Buman MP, Cardon G (2020). World Health Organization 2020 guidelines on physical activity and sedentary behaviour.. Br J Sports Med.

[26] Roberts CE, Phillips LH, Cooper CL, Gray S, Allan JL (2017). Effect of different types of physical activity on activities of daily living in older adults: systematic review and meta-analysis.. J Aging Phys Act.

[27] Lu W, Pikhart H, Sacker A (2019). Domains and measurements of healthy aging in epidemiological studies: a review.. Gerontologist.

[28] Smith L, Soysal P, López-Sánchez GF, Isik AT, Veronese N, Ilie PC (2023). The association between physical activity and urinary incontinence among adults residing in Spain.. Sci Sports.

[29] Nygaard IE, Shaw JM (2016). Physical activity and the pelvic floor.. Am J Obstet Gynecol.

[30] Peinado-Molina RA, Martínez-Vázquez S, Hernández-Martínez A, Martínez-Galiano JM (2023). Impact and influence of urinary incontinence on physical activity levels.. Eur Urol Open Sci.

[31] Faleiro DJA, Menezes EC, Capeletto E, Fank F, Porto RM, Mazo GZ (2019). Association of physical activity with urinary incontinence in older women: a systematic review.. J Aging Phys Act.

[32] Kikuchi A, Niu K, Ikeda Y, Hozawa A, Nakagawa H, Guo H (2007). Association between physical activity and urinary incontinence in a community-based elderly population aged 70 years and over.. Eur Urol.

[33] Kim MM, Ladi-Seyedian SS, Ginsberg DA, Kreydin EI (2022). The association of physical activity and urinary incontinence in US women: results from a multi-year national survey.. Urology.

[34] Hagovska M, Švihra J, Buková A, Horbacz A, Dračková D, Švihrová V (2017). Prevalence of urinary incontinence in females performing high-impact exercises.. Int J Sports Med.

[35] Kuutti MA, Hyvärinen M, Kauppinen M, Sipilä S, Aukee P, Laakkonen EK (2023). Early adulthood and current physical activity and their association with symptoms of pelvic floor disorders in middle-aged women: an observational study with retrospective physical activity assessment.. BJOG.

[36] Parr R, Jones E, Ewen H, Figuers C, Ewen H H (2023). Relationship of sport variables on stress urinary incontinence in nulliparous collegiate athletes.. Journal of Women’s & Pelvic Health Physical Therapy.

[37] Di X, Yuan C, Xiang L, Wang G, Liao B (2024). Association between sitting time and urinary incontinence in the US population: data from the National Health and Nutrition Examination Survey (NHANES) 2007 to 2018.. Heliyon.

[38] Shiue I (2016). Modeling indoor TV/screen viewing and adult physical and mental health: Health Survey for England, 2012.. Environ Sci Pollut Res Int.

[39] Aoki Y, Brown H, Brubaker L, Cornu JN, Faly JO, Cartwright R (2017). Urinary incontinence in women.. Nat Rev Dis Primers.

